# Long-term outcomes of pericardial strip versus prosthetic ring annuloplasty for secondary tricuspid regurgitation by a minimally invasive approach

**DOI:** 10.1186/s13019-021-01723-4

**Published:** 2021-11-21

**Authors:** Giuseppe Nasso, Nicola Di Bari, Giuseppe Santarpino, Marco Moscarelli, Mario Siro Brigiani, Ignazio Condello, Francesco Bartolomucci, Giuseppe Speziale

**Affiliations:** 1 GVM Care & Research, Department of Cardiovascular Surgery, Anthea Hospital, Via Camillo Rosalba, 35/38, Bari, Italy; 2 Department of Experimental and Clinical Medicine, Magna Graecia University, Catanzaro, Italy; 3 Department of Cardiac Surgery, Paracelsus Medical University, Nuremberg, Germany

**Keywords:** Pericardial strip annuloplasty, Secondary tricuspid regurgitation, Autologous pericardial strip, Tricuspid annuloplasty

## Abstract

**Background:**

This study was conducted to compare the outcomes of prosthetic ring versus autologous pericardial strip for the treatment of functional tricuspid regurgitation during left-sided valve surgery by minimally invasive approach.

**Methods:**

From January 2008 and July 2016, autologous pericardial strip (group P-TAP) was used in 109 patients, and prosthetic ring (group R-TAP) in 115 patients. The primary outcomes were long-term overall survival, development of patch degeneration, and significant tricuspid regurgitation recurrence. The second outcome was the assessment of right ventricular functional parameters.

**Results:**

Operative mortality was 1 case (0.9%) in the R-TAP group. At the time of hospital discharge only one patient (0.9%) in the R-TAP group had grade III+ tricuspid regurgitation, and none had grade IV+. Mean follow-up was 94.1 ± 24.5 months. Mild and moderate tricuspid regurgitation recurrence was 3.7% and 4.5% (P-TAP vs. R-TAP groups, *p* = 0.99). Severe regurgitation was observed in 1.8% of cases only in the R-TAP group (*p* = 0.49). There were no reoperations. Late mortality was 3.7% and 5.4% (P-TAP vs. R-TAP groups, *p* = 0.75). Freedom from death, all causes, were comparable among groups (log-rank *p* = 0.45). There were no statistically significant differences between two groups in TAPSE, left ventricular end-diastolic diameter, left ventricular ejection fraction, and left atrial diameter.

**Conclusions:**

Tricuspid annuloplasty using an autologous pericardial strip in patients undergoing minimally invasive surgery is associated to similar long results (survival, late tricuspid regurgitation, and functional echocardiographic parameters) than annuloplasty with a prosthetic ring. In particular, the pericardial strip over time does not develop any degeneration or retraction.

## Background

Secondary tricuspid regurgitation (TR) in the absence of organic lesion of the tricuspid leaflets is the most common form of tricuspid valve disease in developed countries. Over the past decade, a growing body of literature has addressed the criteria to perform concomitant tricuspid annuloplasty in patients undergoing surgery for left-sided heart valve disease [1]. The degree of TR, the tricuspid annular dimension and the coexistence of pulmonary hypertension are currently recognized elements for the establishment of the indication to tricuspid annuloplasty [2–4]. Additionally, it has been ascertained that the implantation of a prosthetic ring for tricuspid annuloplasty improves the freedom from recurrence of TR, the overall and event-free survival at the follow-up compared with suture annuloplasty [5].

The employment of a band of autologous pericardium to perform annuloplasty has been proposed for both the mitral and tricuspid valve [6, 7]. One previous study has suggested this technique is reproducible in the context of tricuspid annuloplasty, and that is associated with better results than suture annuloplasty in terms of long-term freedom from recurrent TR [8]. In another study, the use of autologous pericardium showed results comparable to the use of ring annuloplasty at an average follow-up of 3 years [9].

With the present investigation, we sought to compare the mild and long-term follow-up results of pericardial strip annuloplasty for secondary TR by minimally invasive surgery with those of state-of-the-art prosthetic ring annuloplasty. The purpose is to understand whether the different mechanical properties of these materials may influence the geometrical characteristics of the tricuspid valve and the right ventricle. Furthermore, in an historical period characterized by the increase in social costs and the reduction of hospital resources, it seems important to achieve state-of-the-art clinical results by containing the economic burdens associated with postoperative complications and the use of prosthetic material.

## Methods

### Selection of patients and management of clinical data

Since 2008, all pre-, intra- and postoperative data of patients undergoing cardiac surgery at Anthea Hospital GVM Care & Research, Bari, Italy, are prospectively entered in an electronic database. Such dataset is managed by dedicated personnel and is periodically checked for completeness and consistency. The present report adopts the definitions and recommendations reported in the current guidelines for description of morbidity and mortality after heart valve operations [10]. We queried such database in order to identify the patients who underwent tricuspid valve repair during the period comprised between January 2008 and July 2016 by minimally invasive approach. The repair had to consist in isolated annuloplasty, performed either by the implantation of a semi-rigid prosthetic ring (R-TAP, Ring Tricuspid Annuloplasty) or by annular reinforcement with an autologous pericardial strip (P-TAP, Pericardial Tricuspid Annuloplasty). Patients undergoing suture annuloplasty or any other repair procedure on the tricuspid valve were excluded. The tricuspid valve lesion had to consist in regurgitation graded at least moderate-to-severe (grade III+), and to be secondary in etiology to left-sided heart valve disease. Specifically, TR had to be consequence of the dilatation of the tricuspid annulus and the right ventricle, with ensuing leaflet tethering and malcoaptation, and in the absence of intrinsic leaflet pathology. The complete medical records were retrieved for each patient selected from the database; whether the tricuspid valve lesion as described in the operative and/or pathology reports did not confirm the secondary etiology of TR, the patient was excluded from any further analysis. The condition of preoperative cardiac surgery at the time of tricuspid valve repair was also considered as an exclusion criterion. Similarly, the performance of any other concomitant surgical procedure at the time of tricuspid valve surgery (i.e., coronary revascularization, aortic valve surgery) were exclusion criteria from this study. Only patients undergoing mitral valve surgery were enrolled. Two hundred and twenty-four (224) patients were identified and eligible for the study.

The GVM Care & Research review board approved the study and need for patients’ consent was waived.

### Endpoints

The primary outcomes were long-term overall survival, development of patch degeneration or retraction, and significant tricuspid regurgitation recurrence. The second outcome was the assessment of right ventricular functional parameters in patients previously submitted to tricuspid valve annuloplasty for secondary TR using either a rigid prosthetic ring or an autologous pericardial band by minimally invasive approach.

### Surgical technique

The indication to tricuspid annuloplasty was posed in compliance with the current guidelines [3]. All operations were carried out using general anesthesia, and lung isolation was accomplished using a Carlens double-lumen endotracheal tube. In all patients, the surgical approach was through a 5 cm right anterolateral thoracotomy at the level of the third intercostal space. A right femoral perfusion technique was used in all cases for cardio-pulmonary bypass (CPB). Arterial cannulation was performed with a femoral cannula (Bio-Medicus, Medtronic, Minneapolis, MN), and venous cannulation was accomplished with cannulation of the femoral vein (Sorin LivaNova, London, United Kingdom) and jugular vein (Bio-Medicus, Medtronic). Direct aortic cross-clamp was applied. All patients received normothermic anterograde blood cardioplegia.

The employment of either technique for tricuspid annuloplasty was decided according to surgeon’s preference. In patients receiving P-TAP, a band of autologous pericardium (70 mm in length, 4 mm in width) was harvested prior to establishment of CPB and treated with glutaraldehyde for 3 min. After exposure of the tricuspid valve, the strip was sutured to the tricuspid annulus with interrupted mattress sutures of 2-0 Ethibond suture (Ethicon, Inc, Somerville, NJ) in correspondence of the anterior and the posterior leaflets. Two- to three-mm-interval sutures in the autologous pericardial strip and 5- to 6 mm-interval sutures in the tricuspid annulus can shorter the tricuspid annulus by 50 to 67%, as previously described [8]. When two third of the circumference of the tricuspid valve was reduced to 7 cm, a tricuspid annular diameter would be reduced to about 2.7 to 3.2 cm [8]. In all patients, intraoperative assessment of the valve was done by direct injection of normal saline solution 0.9% in the right ventricle (RV) through the valve in arrested heart. Intraoperative transesophageal echocardiography was used to confirm adequacy of the repair.

In the case of R-TAP, a prosthetic semi-rigid incomplete ring (Cosgrove-Edwards Physio Tricuspid annuloplasty ring^®^; Edwards Lifesciences) was sutured to the tricuspid annulus by standard operative techniques.

Concomitant mitral valve surgery was performed in all patients.

### Long-term follow-up

Post-operative echocardiography before discharge was performed at median 7 days (interquartile range, 6–11 days) in all patients with the exception of a few operative mortality cases. All patients discharged alive from the hospital were followed up at our institution every year after the procedure. Visits included a physical examination with assessment of functional status, 12-lead electrocardiography, and trans-thoracic echocardiography. This was focused on the assessment and quantification of TR, left ventricular ejection fraction, end-diastolic diameters and left atrial diameter. The tricuspid valve regurgitation was classified into four grades: 0 for no regurgitation, I+ for mild regurgitation, II+ for moderate regurgitation, III+ for moderate-to-severe regurgitation, and IV+ for severe regurgitation. Recurrent TR at follow-up was defined as graded at least III+. At follow-up, the right ventricular function was quantitatively assessed by determination of the tricuspid annular plane systolic excursion (TAPSE) [11]. The echocardiographic measurements were performed in compliance with the current guidelines from the European Society of Echocardiography [12].

### Statistical analysis

The assumption of normality of each variable distribution was tested with the Shapiro–Wilk test. Normally distributed variables were reported as the mean ± standard deviation. Categorical variables are reported as number and percentage.

The patients complying with the inclusion/exclusion criteria were divided into two groups according to the technique of tricuspid repair (either R-TAP or P-TAP). Operative mortality was defined as death within the 30th postoperative day. Intergroup comparison was performed using the two-tailed Student’s *t* test and the chi-square test/Fisher’s exact test for continuous and categorical variables, respectively. The Kaplan–Meier method for survival analysis was employed, and the corresponding curves were built. All analyses were performed using R 2.13.2 software (R Development Core Team, Vienna, Austria). The threshold for statistical significance was *p* < 0.05.

## Results

A total of 109 patients who received P-TAP and 115 patients who received R-TAP met the inclusion/exclusion criteria. The baseline characteristics of the study groups are summarized in Table 1; there were no statistically significant differences. Mitral valve surgery (repair or replacement) was performed in all patients. Cross-clamp time and bypass time were comparable among groups (78.6 ± 12.9 min and 79.1 ± 14.4 min, *p* = 0.84, 102.3 ± 9.06 and 104.6 ± 9.3, *p* = 0.16, in the P-TAP and R-TAP groups, respectively, Table 2). Average intensive care unit (ICU) stay was 3.5 ± 2.7 days in the P-TAP group and 3.9 ± 3.8 days in the R-TAP group, *p* = 0.5.

**Table 1:** Baseline characteristics of the study population

Characteristic	R-TAP group N = 115	P-TAP group N = 109	*p*
Age (years)	68.3 ± 10.4	67.6 ± 9.5	0.55
Male gender	61 (53%)	55 (50.4%)	0.80
BMI	22.2 ± 3.4	22.4 ± 3.1	0.27
Preoperative NYHA class III or IV	79 (68.7%)	82 (75.2%)	0.35
*Grade of preoperative TR*			
Severe (IV+)	41 (35.7%)	30 (27.5%)	0.24
Moderate-severe (III+)	74 (64.3%)	79 (72.5%)	0.24
LVEF (%)	50.6 ± 4.1	49.4 ± 4.5	0.17
PASP (mmHg)	47.8 ± 5.2	49.1 ± 4.3	0.13
Renal insufficiency^a^	11 (9.6%)	9 (8.3%)	0.91
Diabetes	18 (15.6%)	13 (11.9%)	0.54
Logistic EuroSCORE I (%)	6.8 ± 2.6	6 ± 2	0.17

Values are presented as the mean ± SD or n (%). There were no missing data

*BMI* body mass index, *NYHA* New York Heart Association, *TR* tricuspid regurgitation, *LVEF* left ventricular ejection fraction, *PASP* pulmunary artery systolic pression

^a^Renal insufficiency: blood creatinine ≥ 2 mg/dL

**Table 2:** Operative data

Variable	R-TAP group N = 115	P-TAP group N = 109	*p*
Bypass time (min)	104.6 ± 9.3	102.3 ± 9.06	0.16
Clamping time (min)	79.1 ± 14.4	78.6 ± 12.9	0.84

Values are presented as the mean ± SD. There were no missing data

*CABG* coronary artery bypass grafting

30-day mortality (defined as death within the 30th postoperative day) was 0.9% in the R-TAP group (1 patient died of cardiogenic shock), while no operative deaths were observed in the P-TAP group.

In hospital mortality (defined as death before discharge) included three patients (2.6%) in the R-TAP group and one (0.9%) in the P-TAP group (*p* = 0.62). In the R-TAP group one patient died of cardiogenic shock, one for septic shock, and one for gastrointestinal complication. In the P-TAP the patient died of cardiogenic shock. The majority of patients who were discharged alive from the hospital presented TR of grade I+; 1 patient (0.9%) in the R-TAP group and none in the P-TAP group presented TR of grade III+, while no patient leaved the hospital with TR graded more than III+. Average hospital stay was 12 ± 7.6 days and 14.1 ± 10.8 days in the P-TAP group and R-TAP group, respectively (*p* = 0.21, Table 3).

**Table 3:** Postoperative variables

Variable	R-TAP group N = 115	P-TAP group N = 109	*p*
ICU stay (days)	3.9 ± 3.8	3.5 ± 2.7	0.5
TR grade 0	25 (21.7%)	21 (19.3%)	0.77
TR grade I+	89 (77.4%)	88 (80.7%)	0.65
TR grade III+	1 (0.9%)	0	0.99
Re-opening for bleeding	1 (0.9%)	1 (0.9%)	0.99
De novo AF	15 (13%)	12 (11%)	0.79
Wound infection	2 (1.7%)	1 (0.9%)	0.99
Respiratory failure or lung complications^a^	9 (7.8%)	7 (6.4%)	0.88
Cardiogenic shock	2 (1.7%)	1 (0.9%)	0.99
Gastrointestinal complication	1 (0.9%)	0	0.99
Renal insufficiency^b^	5 (4.3%)	4 (3.7%)	0.99
Septic shock	1 (0.9%)	0	0.99
Infective endocarditis	0	0	–
Complete AV block	3 (2.6%)	2 (1.8%)	0.99
Hospital stay (days)	14.1 ± 10.8	12 ± 7.6	0.21
30-day mortality	1 (0.9%)	0	0.99
In hospital mortality^c^	3 (2.6%)	1 (0.9%)	0.62

Values are presented as the mean ± SD or n (%). There were no missing data

*ICU* intensive care unit, *AF* atrial fibrillation, *AV* atrio-ventricular

^a^Respiratory failure includes prolonged mechanical ventilation time (> 48 h), need for reintubation, and pneumonia; lung complications include persistent airspace or pneumothorax and significant pleural effusion

^b^Renal insufficiency: blood creatinine ≥ 2 mg/dL

^c^In hospital mortality: defined as death before discharge

Mean follow-up was 94.1 ± 24.5 months (interquartile range, 77–110). Follow-up was conducted every year postoperatively in all patients; 4 cases in each group were lost at follow-up (96.2% and 96.4% completeness in the P-TAP and R-TAP groups, respectively). Four (3.7%) and six (5.4%) patients died during the follow-up in the P-TAP and R-TAP groups, respectively (*p* = 0.75). Among the ten mortalities, 4 were adjudicated to cardiac causes (2 in the P-TAP vs. 2 in R-TAP, *p* = 0.99) and 6 were non-cardiac (two cancer in the P-TAP, two traumatic accident, one cancer and one cerebral hemorrhage in the R-TAP, *p* = 0.68).

There were 4 (3.7%) and 5 (4.5%) cases of recurrence III+ TR in the P-TAP group versus R-TAP group (*p* = 0.99) and 2 case (1.8%) of recurrence III+/IV+ TR in the R-TAP group (*p* = 0.49); no patient received reoperation for TR. The patients with recurrent TR had preoperative low ejection fraction (38%), severe pulmonary hypertension, associated mitral regurgitation and dilated right ventricle.

Four patients were in NYHA III/IV in the P-TAP group versus 7 in the R-TAP group (*p* = 0.54).

**Fig. 1:**
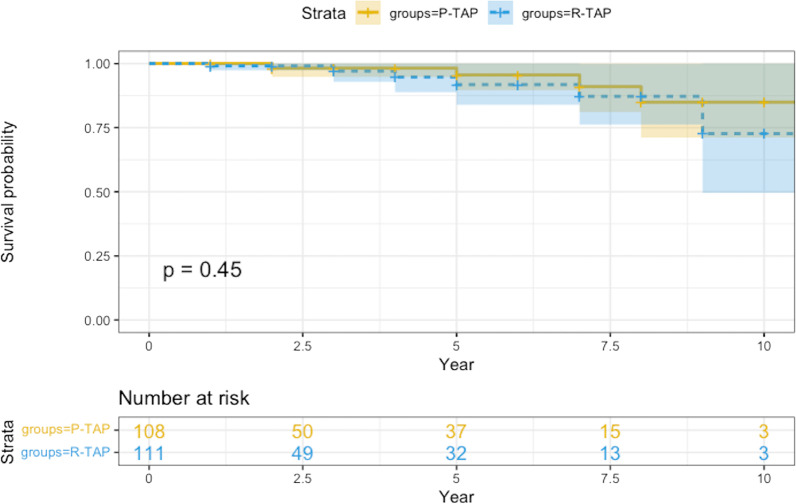
Kaplan–Meier curves: overall survival probability (freedom from death, all causes) in each study group, P-TAP versus R-TAP. *Log-rank *p* value

Figure 1 reports the overall survival probability (freedom from death, all causes) in each study group (Kaplan–Meier methodology). There was no statistically significant difference between the two groups (*p* = 0.45, log-rank test; relative risk P-TA vs. R-TA: 0.62).

Tricuspid valve repair resulted in near-normal morpho-functional parameters of the tricuspid valve (no patch degeneration or retraction) and right/left ventricle in both study groups. We observed no statistically significant differences between two groups at follow-up in terms of average TAPSE (1.36 ± 0.28 cm and 1.39 ± 0.27 in the P-TAP vs. the R-TAP, *p* = 0.71), left ventricular end-diastolic diameter (49.4 ± 4.5 mm and 50.6 ± 4 mm in the P-TAP vs. the R-TAP group, *p* = 0.16), left ventricular ejection fraction (48.8 ± 4.3 and 49.8 ± 3.6 in the P-TAP vs. R-TAP, *p* = 0.16), and left atrial diameter (48.9 ± 4.7 mm and 47.8 ± 5.2 mm in the P-TAP vs. the R-TAP group, *p* = 0.22).

## Discussion

Functional TR is the most common form of TR in Western countries. In recent years, a growing interest has been paid to the treatment of functional TR in patients undergoing surgery for left-sided heart valve disease. Negative prognostic impact of significant TR after left-sided valve surgery, decreased exercise tolerance and poor quality-of-life in patients developing important TR, and the considerable operative risk associated with reoperation for TR, have pushed most surgical groups to a more aggressive policy to the treatment of TR at the time of left-sided heart valve disease [1, 13, 14]. Although several surgical techniques are available for tricuspid repair (including the Kay technique and the ‘clover’ technique) major comparisons have been carried out among the suture annuloplasty (DeVega technique) and the implantation of a rigid open prosthetic ring.

Several studies have indicated that the implantation of a prosthetic ring is associated with better outcomes at follow-up (lower rates of recurrent TR, as well as better survival and event-free survival) than suture annuloplasty [5, 15]. Therefore, use of a prosthetic ring has been progressively accepted as the gold-standard approach. On the other hand, some Authors have previously proposed the use of a strip of autologous pericardium instead of prosthetic material to perform tricuspid annuloplasty [16]. Few clinical data are available over this option; one study compared the P-TAP method to suture annuloplasty, and found better TR recurrence-free survival among the patients treated by pericardial strip8. In another study, the use of autologous pericardium showed results comparable to the use of ring annuloplasty at an average follow-up of only 3 years [9]. We present a longer follow-up with more significance for the evaluation of the pericardial degeneration.

Nonetheless, such study was limited by the comparison of this technique versus suboptimal approaches (suture annuloplasty only). To the best of our knowledge, this study is the first that directly compare the P-TAP technique for secondary TR versus the established prosthetic ring annuloplasty with a mild and long-term follow up in patients undergoing minimally invasive approach.

The first major finding of the present investigation is the comparability of clinical and functional results of the P-TAP versus R-TAP technique in terms of long-term overall survival, development of patch degeneration or retraction, and significant TR recurrence during the long-term follow-up.

We have shown that favourable clinical outcomes can therefore be obtained without the compelling need to use prosthetic materials for tricuspid annuloplasty. This is associated with lower rates of complications at late follow-up (lower risk of prosthetic endocarditis and thromboembolic events), and would determine a containment of hospital costs, since no dedicated consumables would be required. As far as the R-TAP method, the pericardial strip annuloplasty determined no events of atrioventricular block, it can be performed on the beating heart and adds little CPB time to the procedure.

The second finding of our investigation is the comparability among the P-TAP and the R-TAP groups in terms of right ventricular functional parameters (TAPSE) at follow-up echocardiography. Right ventricular function is a major prognostic determinant of recurrence of TR in the long-term [11]. Pericardial strip is much more compliant than a rigid or semi-rigid prosthetic annulus. It has been previously reported in the context of reparative mitral surgery that a pericardial strip for annuloplasty was associated with more favourable mitral annular dynamics and left ventricular function during exercise [17].

Preservation of such morpho-functional unit would be associated with reduced odds of cardiovascular mortality in the long-term follow-up [18]. Long-term durability of the pericardial strip has not been addressed. In the present investigation we observed no cases of strip calcification on echocardiography, strip-related complications or dysfunction. An eight-year follow-up conducted in a previous investigation also indicated optimal durability [8]. In our study tricuspid valve repair resulted in near-normal morpho-functional parameters of the tricuspid valve (no patch degeneration or retraction) and right/left ventricle in both study groups. We observed no statistically significant differences between two groups at follow-up in terms of average TAPSE (1.36 ± 0.28 cm and 1.39 ± 0.27 in the P-TAP vs. the R-TAP, *p* = 0.71), left ventricular end-diastolic diameter (49.4 ± 4.5 mm and 50.6 ± 4 mm in the P-TAP vs. the R-TAP group, *p* = 0.16), left ventricular ejection fraction (48.8 ± 4.3 and 49.8 ± 3.6 in the P-TAP vs. R-TAP, *p* = 0.16), and left atrial diameter (48.9 ± 4.7 mm and 47.8 ± 5.2 mm in the P-TAP vs. the R-TAP group, *p* = 0.22).

## Conclusion

In conclusion, the present study indicates the non-inferiority of the P-TAP technique for tricuspid annuloplasty (based on the use of a strip of autologous pericardium) in patients undergoing minimally invasive approach compared to the current gold-standard annuloplasty with the use of a prosthetic ring.

Performing restrictive tricuspid annuloplasty using an autologous pericardial strip is associated to similar mild and long-term results, follow-up survival, rate of late tricuspid regurgitation and functional echocardiographic parameters than annuloplasty with a prosthetic ring. In particular, the pericardial strip over time does not develop any degeneration or retraction.

### Limitations

This study has several limitations to be noted. First, this study was a retrospective observational study, non-randomized, performed at a single center. Second, the mean follow-up duration of 94 months is sufficiently longer but, given that late TR recurrence tipically matters 10 years after operation, further follow up might be required [19]. Moreover, the use of pericardial strip is for sure cheaper but needs a longer surgical experience on the tricuspid valve to allow a correct valve coaptation.

## Data Availability

All data generated or analysed during the current study are available from the corresponding author on reasonable request.

## Abbreviations

TR: Tricuspid regurgitation
R-TAP: Ring Tricuspid Annuloplasty
P-TAP: Pericardial Tricuspid Annuloplasty
CPB: Cardio-pulmonary bypass
RV: Right ventricle
TAPSE: Tricuspid annular plane systolic excursion
BMI: Body mass index
NYHA: New York Heart Association
LVEF: Left ventricular ejection fraction
PASP: Pulmunary artery systolic pression
CABG: Coronary artery bypass grafting
ICU: Intensive care unit
AF: Atrial fibrillation
AV: Atrio-ventricular
